# Microbiota, Viral Infection, and the Relationship to Human Diseases: An Area of Increasing Interest in the SARS-CoV-2 Pandemic

**DOI:** 10.1097/IM9.0000000000000043

**Published:** 2020-11-04

**Authors:** Matthew D. Moore, Cassandra Suther, Yanjiao Zhou

**Affiliations:** 1 Department of Food Science, University of Massachusetts, Amherst, MA, USA; 2 Department of Medicine, University of Connecticut Health, Farmington, CT, USA.

The current severe acute respiratory syndrome coronavirus 2 (SARS-CoV-2) pandemic and its severe societal burden have underscored the importance of understanding eukaryotic viral infection and its relationship to both acute and secondary health effects.^1,2^ Many viruses, including SARS-CoV-2 in some instances, replicate in the enteric tract or have an indirect relationship with it. A growing area of focus has been to understand the relationship between the gut microbiota and viral infection, as some evidence suggests that certain bacteria or bacterial compositions can promote^3–8^ or inhibit^7,9,10^ viral pathogenesis. The gut microbiota has been implicated in affecting pathophysiology of local gut and many remote organs such as the lungs, liver, and brain.^7^ Although the interaction between viral replication and bacteria in the gut is an exciting growing area of focus, gaps still exist in understanding the effect enteric replication of some viruses has on the gut microbiome and if there are other health consequences of any of those changes.

Human noroviruses are the leading cause of foodborne illness globally, have been demonstrated to interact with enteric bacteria,^3,11–13^ and have been the focus of work showing that their replication can be affected by the gut microbiota. For example, murine norovirus (MNV) replication has been reported to be reduced when the mouse intestinal microbiota was depleted.^3,14^ Pretreatments of mice with antibiotic cocktails showed reduced titers of both acute (MNV-1) and persistent (MNV-3, MNV.CR6) MNV strains.^15^ Conversely, there is some evidence that the gut microbiome may inhibit noroviral infection.^5,6,10^ For instance, a recent report by Barberio et al.^9^ successfully documented the use of fecal microbiota transplantation in a 68-year-old woman who had chronic noroviral diarrhea, with both symptoms and norovirus signal removed after transplantation, along with a notable increase in Bacteroidetes and Proteobacteria and reduction of Firmicutes.^9^ Thus, this supports the possibility that the composition of the gut microbiota can affect norovirus replication in the gut, and may explain some observed differences in disease presentation and susceptibility.^5^

The specific alterations of the overall gut microbiome as a consequence of norovirus infection have also been investigated in both mouse models and humans. MNV-1 infection was found to enhance Firmicutes*/*Bacteroidetes ratios in C57BL/6 mice 5 days post infection. In contrast, MNV-1 and MNV-4 were not found to significantly disrupt the gut microbiota in C57BL/6 mice in a different report.^16^ In another work, a reduction in intestinal *Lactobacillus* strains was found in MNV-1-infected Institute of Cancer Research (ICR) mice, along with an increase in Proteobacteria delta.^15^ In humans, an increase in Proteobacteria (mainly *Escherichia coli*), and decreased Bacteroidetes were found in patients infected with norovirus GII.4, the epidemic genotype.^17^

Although changes in the gut microbiome as a consequence of norovirus infection have been investigated, the implications and mechanisms for these changes have not been as well studied. Some reports suggest that norovirus infection can reactivate the intestinal immune system in microbiota-depleted gut of mice to reduce infection with *Citrobacter rodentium* (a model for enteropathogenic *E. coli*), *Pseudomonas aeruginosa,* and *Enterococcus faecium*^18,19^; suggesting a potential positive effect of viral infection on the gut microbiome. This is thought to be driven by a norovirus nonstructural protein (NS1/2) promoting type I interferons that in turn promotes CCR2-dependent macrophages and interleukin 22.^20^ Additionally, MNV-4 infection in non-obese diabetic mice was found to have protective effects against the development of type 1 diabetes in part due to alteration of the gut bacterial composition by promoting diversity and the Firmicutes*/*Bacteroidetes ratio in mice.^21^ Increases in Firmicutes have been linked to higher small chain fatty acid production, important elements that regulate local and systemic immune response. Bolsega et al.^22^ reported a difference in MNV-induced colitis based on two different defined bacterial flora in an inflammatory bowel disease mouse model, with mice possessing an Altered Schaedler Flora developing colitis but not mice seeded with Oligo-Mouse-Microbiota 12. Although a good deal of exciting findings have been reported with respect to the relationship between noroviruses as well as other food and waterborne viruses (rotavirus, poliovirus) and the gut microbiota,^7^ a number of areas of future study still exist; for example, the potential for other longer term or chronic diseases that occur as a consequence of norovirus-induced alterations in the microbiota. Furthermore, a number of new technologies to monitor the chemical composition of gut activities may help in furthering understanding of the effects and mechanisms of enteric viral replication on the gut microbiome in humans.^23^

Although not traditionally associated with the gut, a number of other viruses have also been shown to replicate in the guts of some individuals, such as influenza and coronaviruses.^24–29^ In fact, SARS-CoV-2 has been detected in the stool of positive patients up to 33 days after negative qPCR tests,^30^ with influenza also detected in the stool of patients.^31^ Direct and indirect interactions between viruses like this and the microbiota have been well-documented.^4,7^ Specifically, many of these interactions appear to be indirect, being facilitated by alterations of the immune system in many cases.^4,7,32^ For example, the gut microbiota has been demonstrated to be capable of assisting immune responses to influenza infection.^32–35^ Alternatively, influenza infection has been shown to reduce microbial diversity in the gut and promote dysbiosis.^36,37^ This alteration is thought in part to be mediated by interferon gamma produced by migration of CCR9^+^CD4^+^ T cells into the small intestine.^38^ Additionally, Type I interferons have also been implicated in the enrichment of Proteobacteria and intestinal dysbiosis as a consequence of influenza infection in a mouse model.^37^ Although very informative, most investigation for influenza has been limited to studying the effects of influenza A in animal models—future work should focus on comparison to other influenza viruses as well as further characterization of shifts in the gut metabolome as a consequence of infection. A recent report by Sencio et al.^39^ found influenza H3N2 and H1N1 both alter the intestinal microbiota and reduce short chain fatty acid production (specifically acetate) locally (gut) and systemically (in blood), leading to reduced immune defenses to other infection.

Undoubtedly, SARS-CoV-2 has been the focus of a considerable amount of research, including some interesting reviews and perspectives suggesting a potential indirect role of the gut microbiota on SARS-CoV-2 infection.^40–42^ However, the effects and interactions of SARS-CoV-2 with the host microbiome remain to be elucidated in-depth. Shen et al.^43^ found the diversity of the microbiota of the lungs of human coronavirus disease 2019 (COVID-19) patients was significantly lower than that of those who were healthy, but found no other significant differences related to the lung microbiota.

A recent report by Zuo et al.^44^ found significant correlations between certain changes in the gut microbiota and SARS-CoV-2 infection in 15 patients with SARS-CoV-2 compared to 15 healthy control patients. Specifically, infection with SARS-CoV-2 was found to result in microbial dysbiosis even after symptoms subsided and virus was presumably cleared. Interestingly, three bacterial species from Firmicutes were positively correlated with SARS-CoV-2 severity, while six Bacteroidetes (four of those in the *Bacteroides* genus) were inversely associated with SARS-CoV-2 load in stool.^44^ Results from the first human trials of multistrain bacterial therapy on COVID-19 patients has been published.^45^ The study included two cohorts of patients, one given traditionally recommended SARS-CoV-2 treatment and the other orally given a cocktail of eight different strains of bacteria including *Streptococcus, Lactobacillus*, and *Bifidobacterium*. Those given the bacterial therapy showed remission, within 72 hours, of diarrhea and other symptoms when compared to the non-supplemented patients.^45^ While including a small sample size, 70 total patients with 28 on bacteriotherapy, this gives evidence to possible viral bacterial interactions with SARS-CoV-2 in the gut. These initial reports are certainly interesting, and future work should focus upon expanding upon them as well as further delving into the mechanistic associations of the microbiome changes and the consequences they have in disease presentation in the near- and long-term. It is important to note that certain host and environmental factors, such as geographical location, race, sex, and age, may play a role in the sensitivity to and/or effect SARS-CoV-2 has on the microbiome of an individual.

In sum, a large amount of researches have both directly and indirectly connected viral infection and the gut microbiota. However, more understanding of viral strain-related differences in humans as well as the metabolomic consequences remains to be investigated for a number of viruses. Emerging breakthrough reports connect SARS-CoV-2 and the human microbiome, but the lifespan of any microbial shifts SARS-CoV-2 causes, their mechanisms, and the metabolomic and health consequences of those shifts remain similarly to be determined.

## Acknowledgments

The authors would like to thank the University of Massachusetts, Amherst for providing funding for this article. The authors would also like to thank the University of Massachusetts Soft Materials for Life Sciences Program for support of C. Suther.
